# Prognostic analysis of cerebral microbleeds in patients with large artery atherosclerotic stroke without thrombolytic therapy

**DOI:** 10.1186/s40001-025-03327-3

**Published:** 2025-11-21

**Authors:** Yuewen Sun, Moxin Luan, Yilong Peng, Chenyang Jin, Xiaoqian Song, Xinbao Yin, Xueping Zheng

**Affiliations:** 1 Department of Geriatric Medicine, The Affiliated Hospital of Qingdao University, No.16 Jiangsu Road, Qingdao, 266001 Shandong China; 2 Qingdao Medical College, Qingdao University, Qingdao, China; 3 Department of Urology, The Affiliated Hospital of Qingdao University, Qingdao, Shandong China

**Keywords:** Cerebral microbleeds, Large artery atherosclerotic stroke, Prognosis, Risk factors, Recurrent cerebral infarction, Mortality, Antiplatelet therapy

## Abstract

**Objectives:**

This study aims to investigate the prognostic implications of cerebral microbleeds (CMBs) in patients with large artery atherosclerotic stroke (LAAS) without thrombolytic therapy, mainly focusing on the association between CMBs and clinical outcomes, such as recurrent ischemic stroke (IS), intracranial hemorrhage (ICH), and all-cause mortality.

**Methods:**

A retrospective cohort study was conducted on 353 LAAS patients (January 2016–October 2021) at the Affiliated Hospital of Qingdao University. All underwent susceptibility-weighted imaging (SWI) and received single or dual antiplatelet therapy. CMBs were classified by location (lobar, deep, and infratentorial) and severity. Kaplan–Meier survival analysis and Cox regression models were used to assess endpoint events.

**Results:**

Among the 353 patients, 147 had CMBs. Significant differences in age, fasting blood glucose levels, and history of hypertension were observed between patients with and without CMBs. Age and hypertension were identified as independent risk factors for CMBs. Patients with CMBs had a significantly higher incidence of recurrent IS and all-cause mortality compared to those without CMBs.

Recurrent IS, ICH, and all-cause mortality were defined as the primary endpoint events in this study. The mean time to endpoint events was shorter in patients with CMBs (59.2 months) compared to those without CMBs (79.1 months). The location of CMBs influenced the prognosis, with deep or infratentorial CMBs associated with higher mortality. However, CMB location does not significantly influence the risk of IS or ICH. Single or dual antiplatelet therapy did not significantly alter the risk of endpoint events in patients with CMBs.

**Conclusions:**

The presence of CMBs, particularly in deep or infratentorial regions, significantly worsens the prognosis of LAAS patients without thrombolytic therapy. Patients with CMBs have a higher risk of recurrent IS and all-cause mortality, but no significant difference in ICH incidence compared to those without CMBs. CMB burden had no differential effect on the efficacy of single antiplatelet therapy (SAPT) or double antiplatelet therapy (DAPT). Further prospective studies are necessary to validate these findings and explore alternative therapeutic strategies.

## Introduction

Large artery atherosclerotic stroke (LAAS) is an ischemic stroke subtype caused by significant stenosis or occlusion of major arteries. LAAS typically occurs in intracranial arteries (such as the anterior cerebral artery, middle cerebral artery, and basilar artery) or extracranial arteries (such as the carotid artery and vertebral artery), where atherosclerotic plaques lead to insufficient blood supply to brain tissue [1]. Major treatments include antiplatelet therapy, high-dose statins, aggressive risk factor management, and lifestyle changes [2, 3]. Notably, LAAS rarely occurs in isolation. Cerebral small vessel disease (CSVD) mainly affects the elderly and refers to a series of pathological changes of small blood vessels in the brain, including white matter hyperintensity (WMH), lacunar infarction, CMBs, and brain atrophy [4]. CSVD commonly coexists with large artery atherosclerosis (LAA), reflecting a significant inter-relationship [5–7]. This may be related to their common vascular damage mechanisms and factors, such as aging, oxidative stress, hypertension, and so on [8]. CMBs are small hemorrhages resulting from damage to the walls of cerebral small vessels. Such vascular injuries may arise from various factors, including hypertension, atherosclerosis, and amyloid angiopathy. As a hallmark of CSVD, the number and spatial distribution of CMBs provide insights into the severity and extent of CSVD-related lesions [9]. Stroke, a major global cause of mortality and long-term disability, imposes an escalating burden worldwide [10]. It represents an acute clinical event stemming from chronic pathological conditions, primarily caused by mechanisms, such as large artery atherosclerosis, cardioembolic events, and CSVD. Due to the extensive application of SWI, the detection of CMBs has become easier. CMBs are increasingly recognized as valuable biomarkers for identifying the nature and severity of underlying CSVD [11, 12]. However, their presence and burden often introduce significant challenges in routine clinical practice [13, 14]. The uncertainty surrounding the risk–benefit balance of such treatments in patients with CMBs highlights the need for careful clinical judgment and further research to optimize management approaches, especially for the number and location of their presence.

Studies have shown that the presence of CMBs is closely associated with an increased risk of poor prognosis [15]. These microbleeds not only reflect the fragility of cerebral vessels but also may indicate a higher risk of subsequent strokes. The presence of CMBs means that pathologically fragile small vessels leak blood, so the study of CMBs is an important part of understanding the risk–benefit balance of antithrombotic therapy in stroke patients [16]. A dual-center retrospective study of Asians indicated that CSVD is associated with stroke recurrence in LAA patients [17]. Ki-Woong Nam et al. analyzed patients with first-ever ischemic stroke classified under the LAA, revealing a dose–response relationship between recurrence risk and the number of CSVD components.

Research has explored the effect of CMBs on the clinical outcomes of ischemic stroke patients [16, 18, 19]. N. Nagaraja et al. found that CMBs are independent predictors of hemorrhagic transformation after intravenous alteplase administration in acute IS [20]. However, the effect of the presence of CMBs on the prognosis of LAAS patients who did not receive thrombolytic therapy remains unclear.

Therefore, we explored the risk factors and underlying mechanisms of CMBs in LAAS patients without thrombolytic therapy, as well as their specific impact on patient prognosis. This study thoroughly analyzed the quantity and distribution of CMBs and their associations with patients' baseline characteristics and clinical outcomes, aiming to provide more scientific evidence to improve the management and treatment of this specific population.

## Methods

### Patients and evaluation

This research aimed to assess whether individuals diagnosed with LAAS who exhibited CMBs faced an elevated likelihood of experiencing poor events compared to those without CMBs, and to determine if different antiplatelet strategies influenced their long-term outcomes. This retrospective analysis encompassed a continuous cohort of acute LAAS patients who underwent SWI imaging and were hospitalized at the Affiliated Hospital of Qingdao University, China, from January 2016 to October 2021. At admission, baseline demographic information and clinical risk factors—including age, sex, and stroke-related conditions, such as hypertension, diabetes, and history of smoking or alcohol consumption—were recorded. To eliminate potential confounding effects of anticoagulants, only patients with acute IS who exclusively received antiplatelet therapy were included. Eligible participants were those admitted within 48 h of symptom onset and diagnosed with LAAS based on the TOAST classification criteria [2].

### Neuroimaging analysis

CMBs were characterized as lesions with a diameter smaller than 10 mm, exhibiting very low signal intensity on SWI, appearing as homogeneous, round focal areas distributed across the brain [15]. The anatomical classification of CMBs was divided into strictly lobar, deep, and infratentorial categories. Patients presenting with one or more CMBs confined to the cerebral cortex, subcortical junction, or insula were classified as having strictly lobar CMBs. Those with CMBs located in deep regions, including the basal ganglia, thalamus, corona radiata, internal capsule, and external capsule, or in infratentorial areas, such as the brainstem and cerebellum, were categorized as having deep or infratentorial CMBs regardless of whether lobar CMBs were present. The assessment of CMBs on SWI was independently conducted by two neurologists who were blinded to the clinical data of the participants. If there are significant differences, a third expert will be invited to make a ruling. The inter-rater agreement for CMB assessment was substantial (κ = 0.82).

### Antiplatelet therapy

The choice of antiplatelet therapy was determined by the attending physician at the time of hospital admission and included options, such as aspirin, clopidogrel, ticagrelor, dipyridamole, and cilostazol. DAPT was prescribed for at least 3 weeks, followed using a single antiplatelet agent; the actual application time of the DAPT depended on the decision of the clinician. SAPT is prescribed for at least 3 weeks as well.

### Outcome measure and follow-up

The primary endpoints were recurrent IS, ICH, and all-cause mortality. Follow-up was conducted via telephone to monitor symptoms, medication use, and hospital readmissions post-discharge. All patients underwent follow-up for a period ranging from a minimum of 2 years to a maximum of 7 years after discharge.

### Statistical analyses

All statistical analyses were performed using the Statistical Package for the Social Sciences version 26.0 (SPSS, Chicago, IL, USA). Continuous variables were presented as mean ± standard deviation and compared using the independent *t* test. Categorical variables were compared using the chi-square test or Fisher’s exact test as appropriate. Differences in outcomes were analyzed using Cox regression models for survival. Hazard ratios (HRs) and 95% confidence intervals (95% CIs) were calculated. Cumulative event-free frequencies were calculated using the Kaplan–Meier method, and the significance level was set to P = 0.05.

### Standard registrations and ethical approvals

Ethical approval for this study was obtained from the Institutional Review Board of the Affiliated Hospital of Qingdao University (QYFY WZLL 28568). Due to the retrospective study design and substantial sample size, informed consent was waived.

## Results

### Baseline characteristics of the study participants

**Fig. 1:**
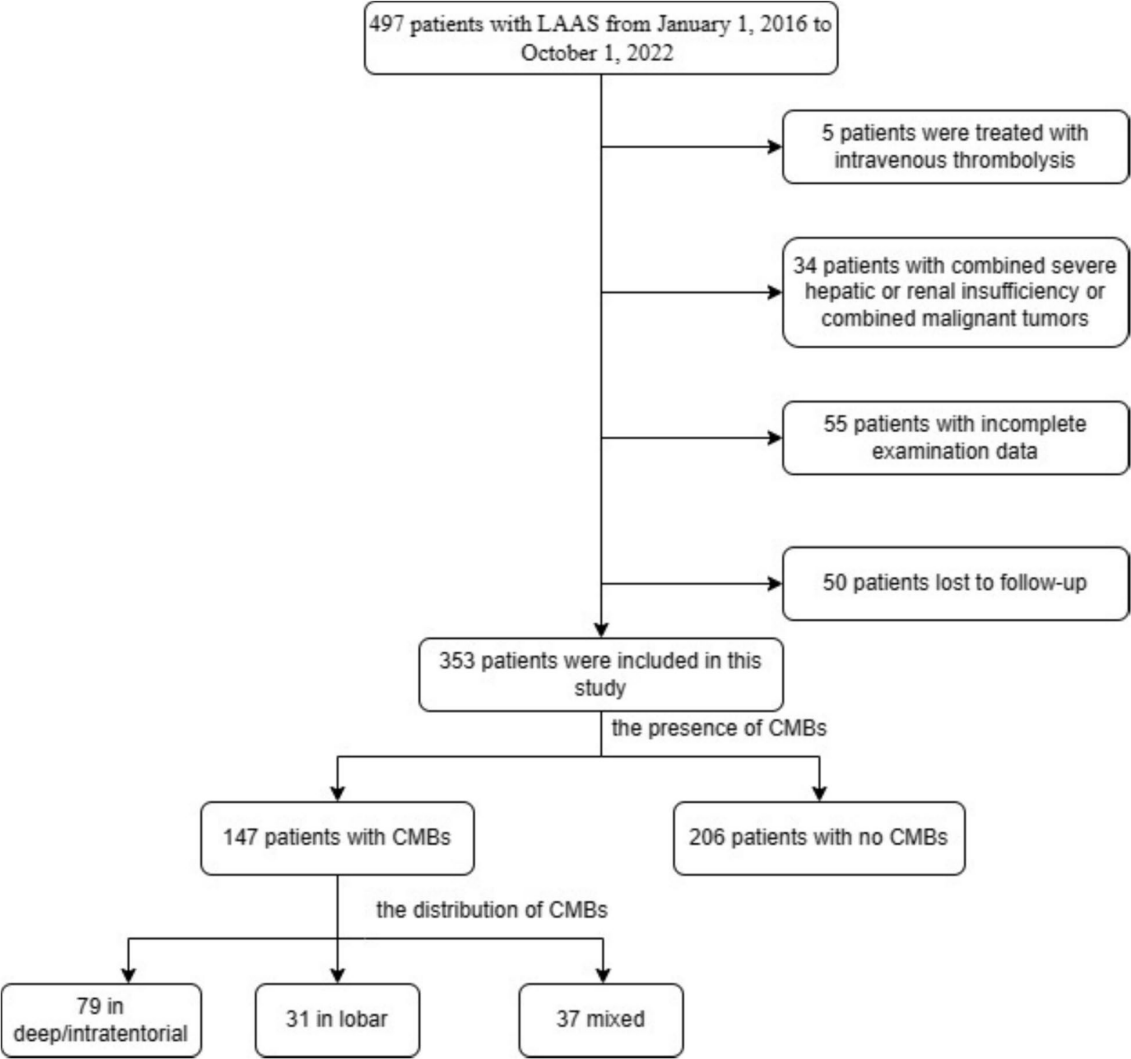
Research flowchart

A total of 353 patients diagnosed with LAAS were retrospectively included from the Affiliated Hospital of Qingdao University (Fig. 1). Baseline characteristics, including demographics, vascular risk factors, laboratory parameters, and neuroimaging data, were collected. Among these patients, 147 (41.6%) presented with CMBs, while 206 (58.4%) had no evidence of CMBs. The distribution of lesion locations in the CMBs group was as follows: 79 patients had lesions in deep or infratentorial locations, 31 in the cerebral lobes, and 37 in mixed locations. CMB burden was stratified as mild (1–5), moderate (6–10), and severe (> 10) in 90, 19, and 38 patients, respectively. Patients with LAAS were divided into two groups based on the presence or absence of CMBs for comparison of general conditions and clinical characteristics (Table 1). Statistically significant differences between the two groups were observed in age (*P* = 0.026), fasting blood glucose (*P* = 0.046), and history of hypertension (*P* = 0.003). No significant differences were found in the history of diabetes, coronary artery disease, blood lipids, creatinine, urea nitrogen, homocysteine, and BMI between the two groups. No significant differences were observed in other vascular risk factors, biochemical indicators, or lifestyle-related variables.

**Table 1:** Baseline characteristics

	CMBs group (n = 147) (%)	No CMBs group (n = 206) (%)	*P*
Age	68.26 ± 11.13	65.50 ± 11.60	0.026
Sex(male)	93(63)	149(72)	0.091
TG	1.34 ± 0.87	1.50 ± 0.78	0.076
TC	4.31 ± 1.34	4.39 ± 1.13	0.547
LDL	2.57 ± 1.01	2.61 ± 0.85	0.663
BUN	5.87 ± 2.94	5.56 ± 2.85	0.318
Cr	82.94 ± 25.48	85.53 ± 26.53	0.360
FBG	6.00 ± 1.96	6.48 ± 2.61	0.046
Homocysteine	13.10 ± 5.13	12.91 ± 4.42	0.709
CRP	7.54 ± 18.65	7.72 ± 22.47	0.938
BMI	24.5 ± 2.9	25.1 ± 3.9	0.281
Hypertension	108(73)	119(57)	0.003
Diabetes	52(35)	79(38)	0.646
coronary heart disease	34(23)	41(19)	0.549
Current smoker	54(37)	88(42)	0.308
Alcohol abuse past year	36(24)	51(25)	1.000
White matter demyelination	66(45)	101(51)	0.503

### Efficacy outcomes

**Fig. 2:**
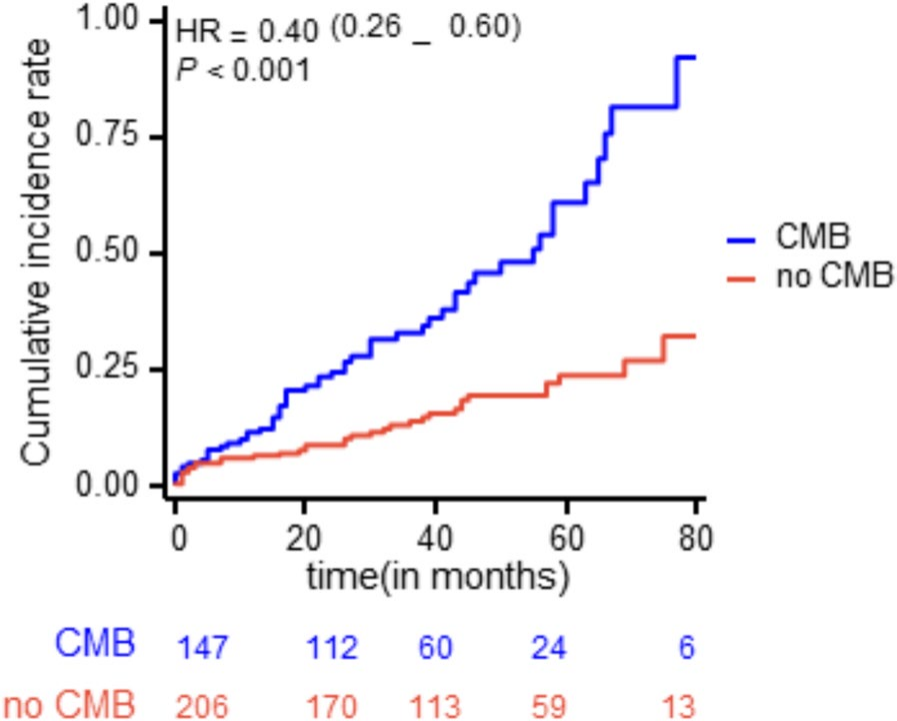
Kaplan–Meier survival curves for patients with or without CMBs

We evaluated the times to events of two groups of patients using the Kaplan–Meier survival analysis. The mean time for the entire sample was 69.2 months. The mean time to endpoint events for the group with CMBs was 59.2 months, whereas for the group without CMBs, it was 79.1 months. In addition, the cumulative incidence of endpoint events was notably higher in the group with CMB (Fig. 2).

In this study, we considered recurrent IS, ICH, and all-cause mortality as endpoint events. During the follow-up period, a total of 91 patients experienced these endpoint events, with 55 cases in the CMB group and 36 cases in the non-CMB group. Univariate Cox regression analysis of the two groups (Table 2) showed significant differences in the recurrence of cerebral infarction (hazard ratio HR=2.711, confidence interval CI 1.481–4.963; *P*=0.001) and all-cause mortality (HR=2.603, CI 1.373–5.038; *P*=0.004), indicating a higher probability of endpoint events in the CMB group. However, no significant difference was observed between the two groups concerning ICH. Overall, there was a statistically significant difference in the risk of endpoint events between patients with and without CMB (HR=2.414, CI1.586–3.676; *P*<0.001).

**Table 2:** Impact of CMBs on outcomes

	CMBs group (*n* = 147)	No CMBs group (*n* = 206)	HR (95%CI)	*P*
Recurrent IS	28	17	2.711(1.481–4.963)	0.001
ICH	3	4	1.216(0.281–5.659)	0.763
All-cause mortality	24	15	2.630(1.373–5.038)	0.004
Total	55	36	2.414 (1.586–3.676)	0.000

CMBs located in different regions may be caused by different pathological reasons. CMBs in the cortical regions might be due to cerebral amyloid angiopathy (CAA), while deep or infratentorial CMBs may be caused by arteriosclerosis. Therefore, based on the results of the SWI examinations, those with CMBs were divided into cortical, deep, infratentorial, and mixed groups. As shown in Table 3, compared to the deep/infratentorial group, the cortical group showed a significantly reduced risk of all-cause mortality (HR = 2.555, 95%CI 1.035–6.307; *P* = 0.042), but there was no significant difference in the risk of recurrent IS or ICH; the mixed group showed no significant difference in the risk of recurrent IS, ICH, or all-cause mortality. It can be intuitively seen from Table 3, patients with higher numbers of CMBs demonstrated an increasing trend in the incidence of composite endpoint events.

**Table 3:** Impact of distribution and number of CMB on outcomes

			Recurrent IS		ICH		All-cause mortality	
	*n*		HR (95%CI)	*P*	HR (95%CI)	*P*	HR (95%CI)	*P*
Distribution of CMB								
Deep/infratentorial	79		1		1		1	
Lobar	31		2.041 (0.870–4.785)	0.101	2.868(0.178–46.154)	0.457	2.555 (1.035–6.307)	**0.042**
Mixed	37		1.076 (0.408–2.838)	0.882	2.411(0.150–38.720)	0.534	1.235 (0.418–3.644)	0.702
Number of CMB								
0	206		1		1		1	
1–10	109		2.940 (1.558–5.549)	** < 0.001**	0.574(0.064–5.154)	0.620	2.345 (1.149–4.784)	**0.019**
> 10	38		2.123 (0.836–5.387)	0.113	3.119(0.570–17.081)	0.190	3.443 (1.456–8.141)	**0.005**

Bold values indicate statistically significant associations (P <0.05).

_Footnote: Bold values indicate statistically significant associations (P<0.05)._

**Fig. 3:**
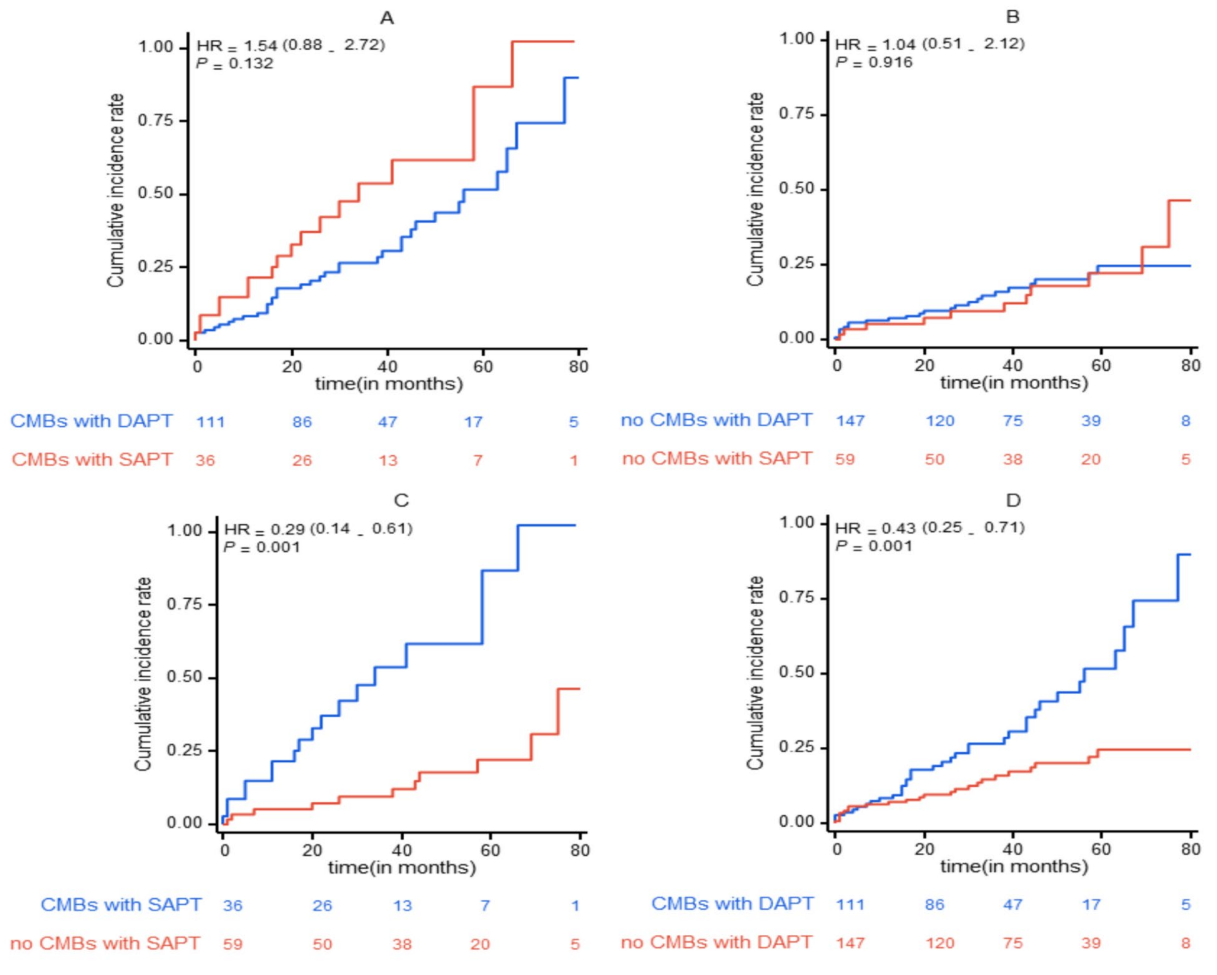
Comparisons of the cumulative incidence of the primary endpoint

Patients with LAAS were divided into 95 in the SAPT group and 258 in the DAPT group according to the antiplatelet therapy regimen. Figure 3 shows that short-term application of SAPT or DAPT did not affect the risk of endpoint events in patients with comorbid CMBs (*P* = 0.132), as well as in patients without comorbid CMBs (*P* = 0.916). The risk of composite endpoint events (including recurrent cerebral infarction, ICH, and all-cause death) was significantly higher in patients with either short-term application of DAPT or SAPT, both in combination with CMBs, compared with patients without CMBs (*P* = 0.001).

Figure 3 shows the pairwise comparisons estimate of cumulative incidence for the primary endpoints—a composite of recurrence IS, ICH, and all-cause mortality among the four groups. (A), the CMBs with SAPT group and CMBs with DAPT group; (B), the no CMBs with SAPT group and CMBs with DAPT; (C), the no CMBs with short-term SAPT group and CMBs with SAPT group; (D), the no CMBs with dual DAPT group and CMBs with DAPT group.

**Fig. 4:**
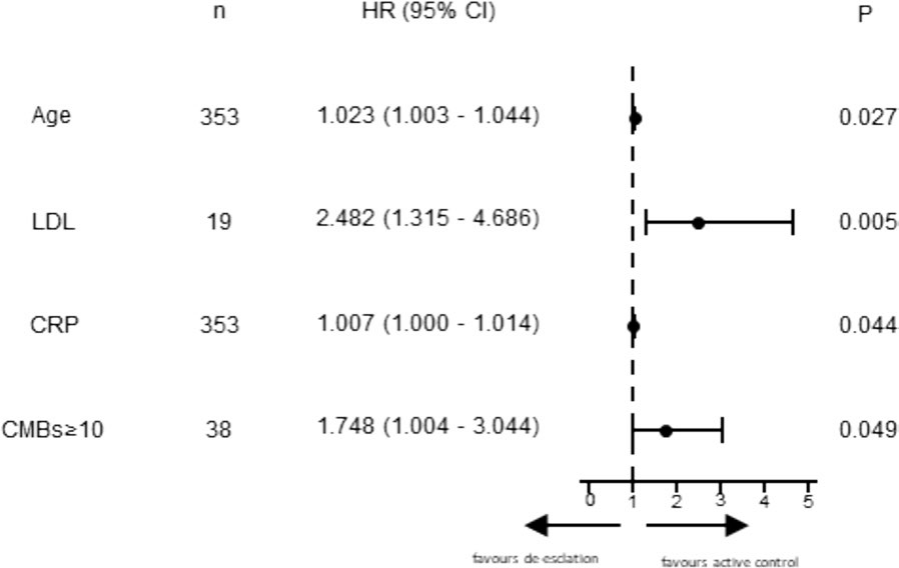
Forest plot showing cox regression models

Multivariate Cox proportional hazards regression analysis (Fig. 4) identified four independent predictors of major endpoint events in this cohort. The results show that increased age (HR = 1.023, 95% CI 1.003–1.044; *P* = 0.027), dyslipidemia (HR = 2.482, 95% CI 1.315–4.686; *P* = 0.005), CRP levels (HR = 1.007, 95% CI 1.000–1.014; *P* = 0.044), and having 10 or more CMBs (HR = 1.748, 95% CI 1.004–3.744; *P* = 0.049) significantly increased the risk of major endpoint events.

## Discussion

This study highlights the association between CMBs and adverse clinical outcomes, including recurrent IS, ICH, and all-cause mortality while addressing the mechanisms, prognostic implications, and therapeutic challenges associated with CMBs in LAAS. This study findings indicate that the presence of CMBs, especially in deep or infratentorial regions, is linked to an elevated risk of adverse clinical outcomes, including recurrent IS, ICH, and all-cause mortality. The hazard ratio for all-cause mortality is notably higher in patients with deep or infratentorial CMBs compared to those with lobar CMBs. In terms of treatment, the results indicate that neither DAPT nor SAPT significantly affected the risk of end-point events in CMB patients.

We observed significant differences in baseline characteristics, including age, fasting blood glucose, and history of hypertension, between patients with and without CMBs, supporting established associations between these risk factors and CMB development. A study has also reported positive correlations between hypertension, prior cerebral infarction, and homocysteine levels with CMB severity, while total cholesterol showed a negative correlation [21]. The differences between our empirical findings and those reported above suggest that research on different aspects of risk factors in patients with CMB needs to continue.

Studies have established an association between CMBs and an elevated risk of stroke, IS, brain hemorrhage, Alzheimer's disease, dementia, and death [22]. In a study of 2,076 Chinese patients with intracranial atherosclerosis, the co-occurrence of intracranial atherosclerosis and WMH in patients was found to predict poor functional outcomes at 1 year, but not stroke recurrence [23]. This finding implies that CMBs, as a significant imaging marker of CSVD, may potentially correlate with clinical prognosis.

CMB characteristics and implications also differ across other stroke subtypes. The occurrence of CMBs in stroke patients varies based on the ischemic stroke subtype [24]. For instance, the rate of CMBs in patients with cerebral infarction resulting from large LAA ranges from 9% to 41.3% [25, 26]. The microbleeding rate was higher in patients with lacunar infarction and atherosclerotic thrombotic infarction. The microbleeding rate of patients with cardiogenic embolic infarction is low [27]. The location of CMBs was primarily in deep regions for patients with small vessel occlusion (SVO) and LAA, while cardioembolic stroke patients exhibited a higher frequency of CMBs in the lobar areas compared to those with SVO and LAA. Cardioembolic stroke, on the other hand, is less commonly associated with deep CMBs but may present with cortical microbleeds due to embolic events and distal vessel occlusion, raising the risk of hemorrhagic transformation under anticoagulation therapy [28]. In ischemic stroke patients with atrial fibrillation, the presence of CMBs was linked to a higher risk of symptomatic ICH during follow-up, but no association was found with the recurrence of IS [29]. At present, there have been some studies to explore the co-occurrence of CMBs in different diseases, but the study on the impact of the co-occurrence of CMBs on the prognosis of patients is not clear enough, which is the direction we can strive for in the future.

Now, some studies have found that the effects of CMBs on patients may vary depending on the location of the lesion. It has been found that patients with CMBs in the cortical region are more associated with an increased risk of cognitive impairment and have a greater impact on patients' functional outcomes [30, 31]. However, patients with CAA-associated CMBs have a significantly higher risk of cerebral hemorrhage compared to those with LAAS. CAA is mainly associated with cortical CMBs resulting from amyloid deposition, which compromises vessel integrity and heightens the risk of ICH, balanced by the risk of both ischemia and bleeding, complicating treatment decisions [32, 33]. Similarly, it has been found that patients with basilar branch atherosclerosis have higher WMH severity in the periventricular region (but not in the subcortical region) compared to LAA [34]. Unfortunately, due to the limitations of sample size and study design, this study did not distinguish LAAS cases based on lesion location. Future larger and more diverse studies may provide a more detailed analysis of the relationship between LAA and CMBs, providing new insights into personalized management strategies for LAAS patients.

Based on our experimental results and existing theoretical studies, we can make the following conjectures. On one hand, this may be due to the unique structural and functional characteristics of blood vessels in these areas. The occurrence of CMBs in these regions typically signifies more severe small vessel lesions. These lesions can readily lead to significant neurological damage, necessitating prolonged bed rest for patients. This immobility heightens the risk of complications, including lung infections and deep vein thrombosis, thereby deteriorating patient prognosis [12]. On the other hand, striking a balance between bleeding and ischemic risk is more challenging for deep and sub-tentorial CMB patients receiving antiplatelet or anticoagulant therapy. Blood vessel rupture and hemorrhage in these regions can result in severe outcomes, including life-threatening brainstem hemorrhages, particularly when large atherosclerosis or other conditions necessitating antiplatelet therapy for ischemic stroke prevention are present. Complicated aortic lesions were linked to deep and widespread CMBs, whereas previous anticoagulant use was associated with lobar CMBs [35]. This complexity in treatment decisions significantly impacts patient prognosis.

Regarding APT, our research did not observe any notable difference in endpoint events between short-term DAPT and SAPT in CMB-positive LAAS patients, echoing the findings of Aoki et al. However, the risk–benefit balance of DAPT versus SAPT remains complex. Aoki et al. found that the combination of cilostazol and aspirin did not show a correlation with a higher risk of ICH compared with aspirin alone, and there were no significant differences in effectiveness or safety results between treatment groups [36]. However, in a research involving individuals with acute IS combined with CMBs, the occurrence of ICH was more frequent in the DAPT group compared to the CMBs group, suggesting that DAPT could offset the risk of CMBs leading to recurrence of IS, but increased the risk of ICH [37]. We can make the assumption that short-term antiplatelet therapy may not be sufficient to observe a considerable impact on the outcomes of patients with CMBs, especially in the non-acute phase. It is also possible that safety may have been adequately considered in treatment selection and dose adjustment so that even DAPT does not increase bleeding risk.

Although this study offers important insights, there are several limitations to consider. The retrospective nature of the study may lead to selection bias, and reliance on SWI for detecting CMBs, though highly sensitive, may not capture all microbleeds. Future prospective studies with larger cohorts and longer follow-up periods are needed to validate these findings and explore the effectiveness of alternative therapeutic strategies. In addition, Werring et al. highlighted that the detection and classification of CMBs might vary across studies, potentially affecting the comparability and consistency of results [38]. Thus, future research should standardize detection and classification methods to ensure the reproducibility and comparability of results.

## Conclusion

Our findings suggest that CMBs, especially in deep or infratentorial regions, are associated with worse outcomes in LAAS patients without thrombolysis. CMBs may serve as valuable markers for risk stratification. Furthermore, standard antiplatelet therapies may not adequately address the risks in these patients, necessitating a more personalized approach to treatment. Future research should focus on elucidating the mechanisms driving the differential impact of CMBs and developing targeted interventions to improve patient outcomes.

## Data Availability

The data sets used and/or analyzed during the current study are available from the corresponding author on reasonable request.

## Abbreviations

CMBs: Cerebral microbleeds
LAAS: Large artery atherosclerotic stroke
IS: Ischemic stroke
ICH: Intracranial hemorrhage
SWI: Susceptibility-weighted imaging
SAPT: Single antiplatelet therapy
DAPT: Double antiplatelet therapy
CSVD: Cerebral small vessel disease
WMH: White matter hyperintensity
LAA: Large artery atherosclerosis
HRs: Hazard ratios
95% Cis: 95% Confidence intervals
CAA: Cerebral amyloid angiopathy
